# Surgical Management for Refractory Bleb Dysesthesia

**DOI:** 10.1155/2020/7570454

**Published:** 2020-08-03

**Authors:** Agnieszka Dyrda, Alfonso Anton, Juan Pablo Figueroa-Vercellino, Marta Pazos

**Affiliations:** ^1^Institut Català de la Retina (ICR), Barcelona, Spain; ^2^Universitat Internacional de Catalunya (UIC), Barcelona, Spain; ^3^Institut Clínic d'Oftalmologia, Hospital Clínic de Barcelona, Universitat de Barcelona, Barcelona, Spain

## Abstract

**Purpose:**

To present long-term results of modified bleb-limiting conjunctivoplasty as a successful treatment for intractable bleb dysesthesia and to review the literature on the surgical management of dysesthetic bleb.

**Methods:**

Consecutive case series and literature review. We present four cases of surgically reduced painful blebs. Our technique consisted of the following steps: (1) conjunctival, radial incision to the bare sclera in the desired limit of the bleb; (2) suturing with buried, interrupted sutures at the nearest edge of the filtering bleb; (3) lower limbal peritomy including unwanted area of the extended bleb; (4) dissection and removal of the underlying fibrous tissue when present; (5) conjunctival and resorbable sutures. In addition, a systematic literature review was performed. Only reports presenting outcomes of surgical treatment of bleb dysesthesia after filtering procedure were included in review.

**Results:**

Four eyes were included consecutively in the study in a period of 4 years. On average, they developed circumferential bleb dysesthesia 9.3 ± 4.7 months after uneventful combined phacotrabeculectomy with Mitomycin C as primary procedure. Surgical reduction was decided after failure of lubricants in controlling ocular discomfort. Two cases showed a dense fibrous tissue beneath the conjunctiva that was excised to ensure filtration. In all cases, a rapid disappearance of symptoms with very good aesthetic and functional outcome was observed. After 12-month follow-up, patients remained asymptomatic and maintained intraocular pressure of 10.7 ± 1.2 mmHg without treatment. A systematic review of the literature obtained 15 eligible case series (*n* = 123) with rates of success within 46–100%, favoring less aggressive approaches to reduce bleb size.

**Conclusion:**

Bleb dysesthesia is a rare complication of filtering glaucoma surgery. This modified bleb-limiting conjunctivoplasty technique (with removal of subjacent fibrous tissue if present) is able to target the underlying etiology providing ocular discomfort relief while maintaining bleb function and may be considered as first-choice surgical treatment.

## 1. Introduction

In 1977, Cohen et al. proposed classifying dysfunctional filtering blebs into three basic categories: underfiltration, overfiltration, and excessive size [1]. Bleb dysesthesia is an uncommon complication of glaucoma filtration surgery in which a well-functioning, but large filtering bleb with adequate intraocular pressure (IOP) control causes ocular discomfort [2–4] due to the effect of interrupted tear film distribution over the bleb and cornea [5].

There are many described methods for managing dysesthesia. Intensive surface lubrication is universally used. Other nonsurgical methods that stimulate scarring (i.e., laser, cryocoagulation, etc.) can lead to IOP increase [2]. Planned surgery, in the case it is needed, should ideally be effective in not only relieving the symptoms, but also maintaining bleb function.

## 2. Objective

The objective of this study is to present the characteristics and outcomes of consecutive cases of bleb dysesthesia refractory to medical lubricant treatment that underwent bleb-limiting conjunctivoplasty surgery from 2015 to 2019 and, secondarily, to perform an extensive online literature search to identify other studies reporting on the surgical management of bleb dysesthesia.

## 3. Methods

This case series is a prospective study of consecutive patients that developed refractory bleb dysesthesia after successful filtration surgery in a 4-year time period. This study was approved by the Ethical Committee and was conducted in accordance with the tenets of the Declaration of Helsinki. Written informed consent was obtained from all the participants.

### 3.1. Patient Examination

All the participants underwent a thorough ophthalmic evaluation before and 1 day, 1 month, 6 months, and 1 year after conjunctivoplasty: best-corrected visual acuity (BCVA) in Snellen (imperial) scale, standard automated perimetry with Swedish Interactive Threshold Algorithm (SITA) standard strategy, program 24–2 of the Humphrey Field Analyzer (Carl Zeiss Meditec, Germany), Goldman applanation tonometry, slit-lamp biomicroscopy with IBAGS scale [6] for bleb evaluation, gonioscopy, fundoscopy with vertical cup to disc ratio estimation by two glaucoma specialists (MP, AA), and HD-OCT imaging using the standard peripapillary protocol (retinal nerve fiber layer (RNFL) thickness) of Cirrus OCT (Carl Zeiss Meditec, Germany).

### 3.2. Surgical Technique

The conjunctivoplasty surgery consisted of the following (video attached):

- One radial conjunctival incision through the bleb at 10 : 30 (left eye) or 1 : 30 (right eye) position down to bare sclera in the desired limit of bleb (to decrease the size of bleb) (all 4 eyes)
- Suturing with buried, interrupted sutures, the nearest edge of the filtering bleb (all 4 eyes)
- Lower limbal peritomy including unwanted area of the extended bleb (all 4 eyes)
- Dissection of conjunctiva from underlying connective tissue (all 4 eyes), excision of subconjunctival fibrous tissue (in 2 eyes with thick bleb)
- Repositioning of conjunctiva with interrupted resorbable sutures attached to sclera (all 4 eyes)

### 3.3. Variable Outcomes

Ocular discomfort was expressed freely by patients when dysesthetic bleb was diagnosed and then asked again after conjunctivoplasty at each postoperative visit. Postoperative data including IOP, bleb appearance, VF results, and OCT results was collected at the first 24 hours and then 1, 6, and 12 months after bleb reductive surgery.

### 3.4. Literature Search

The search for studies to review was performed on the Medline and Embase databases up to March 2020, with no language limits, combining 2 groups of terms: [bleb] AND [dysesthesia]. All terms were used as MeSH and as free terms. All articles were revised by 2 investigators independently (AD, MP). Considering the rarity of this condition, articles were included if they were prospective or retrospective case studies presenting outcomes of surgical treatment of circumferential dysesthetic blebs after filtering procedure. Additionally, data of surgical management and outcomes were extracted from the article body.

## 4. Results

Four consecutive patients with refractory bleb dysesthesia were included in this study during the 4-year time period. We present their baseline characteristics and postoperative outcomes of a modified bleb-limiting conjunctivoplasty procedure and an overview of previously described cases in the literature.

### 4.1. Case Series

The study group consisted of 4 eyes of 2 women and 2 men whose ages ranged from 69 to 81 years (mean ± SD, 72.7 ± 7.4 years). All patients were from European descent and had primary open-angle glaucoma treated with combined phacoemulsification and trabeculectomy with Mitomycin C (MMC) as primary procedure (see Table 1). During the early postoperative period, additional treatment with 5-fluorouracil (5-FU) and argon laser suturolysis was needed to achieve IOP control in 3 of the 4 eyes. Three months postoperatively, bleb was diffuse and elevated in all cases, with IOP under target.

**Figure 1:**
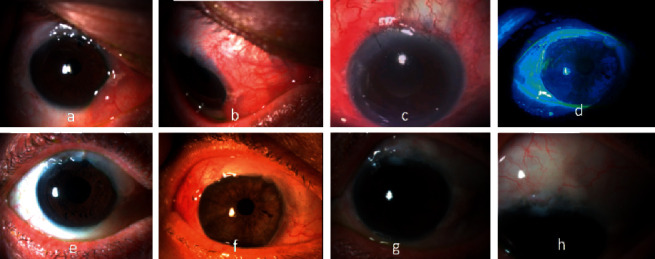
Dysesthetic bleb before surgical treatment: nasal (a–c) or temporal (d) extension, hyperemia (a, b) and Dellen (c, d). 1^st^ month (e, f) and 12^th^ month (g, h) after bleb-limiting conjunctivoplasty.

After a mean of 9.3 ± 8.5 months from primary filtration procedure, the 4 cases developed circumferential, prominent blebs with a mean extension of 168° and nasal (*n* = 3) or temporal (*n* = 1) displacement (Figure 1 (a–d)) associated with ocular discomfort with intense foreign body sensation. Main characteristics of bleb dysesthesia are shown in Table 1. Two patients presented with a corneal Dellen (50%).

Symptomatic relief was not referred by any patient, although intensive lubrication was used, so bleb-limiting conjunctivoplasty was then indicated and performed. No intraoperative complications were noted. In all cases, immediate relief and cosmetic improvement were observed within the first-month follow-up (Figure 1 (e, f)). Although bleb size was reduced intentionally, long-term significant IOP elevation was not observed in any case. Only one eye experimented a self-limited IOP spike (IOP of 26 mmHg) one day after surgery that resolved spontaneously within 24 hours (Case 4). Average IOP was 16.3 ± 7.1, 10.5 ± 2.5, 11.5 ± 1.9, and 11.5 ± 1.9 mmHg for postoperative day 1 and months 1, 6, and 12 after surgery, respectively. None of the cases needed any additional glaucoma medication or surgery after 1 year of follow-up. Visual acuity, visual field, and OCT did not show significant deterioration after 1 year. Surgical outcomes are listed in Table 1.

### 4.2. Literature Search

**Figure 2:**
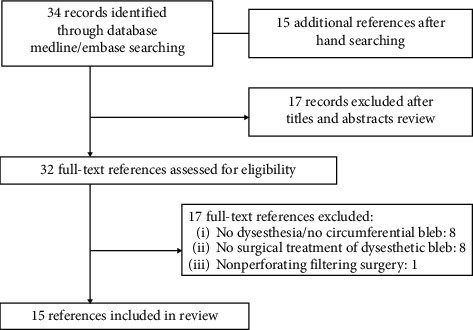
Flowchart showing literature search.

The complete flowchart is presented in Figure 2. Our search yielded 34 records and provided 15 additional titles after hand-searching their related references (total of 49 studies). Of these, 17 records were excluded directly after titles and abstracts evaluation. Two reviewers (AD, MP) independently evaluated these 32 studies. After this full-text assessment, 17 further records were excluded as they did not meet our study inclusion criteria. Finally, this review of available literature resulted in a total of 15 articles [3–5, 7–18] containing 123 cases of surgical management of circumferential, dysesthetic blebs after penetrating filtering glaucoma surgery (Table 2). We assessed all eligible studies, extracted characteristics, and summarized key findings.

## 5. Discussion

Bleb dysesthesia is a rare complication of glaucoma surgery in which a well-functioning bleb with well-controlled IOP causes ocular discomfort typically related to an increase in its size [1–4].

Although bleb dysesthesia etiology is still not fully elucidated, two potential productive mechanisms have been described: first, a hypocellular tissue response probably related to the use of antimetabolites creating an avascular bleb that then reaches a larger size [2]; second, a connective tissue hypertrophy caused by an extensive scarring reaction in a susceptible eye [2]. In this direction, histology studies have reported a dense cellular connective tissue even within functioning blebs [2]. It is suggested that such tissue, if presented under the conjunctiva, encourages the formation of dysesthetic blebs physically contributing to the bleb height, and facilitating the formation of a subconjunctival reservoir for aqueous humour beneath its smooth surface [4]. In our study, MMC was used intraoperatively in all 4 cases at the time of the first filtering procedure, and 3 of the 4 eyes received one or more 5-FU postoperative injections to modulate filtration. In fact, two blebs that had undergone application of both antimetabolites showed mostly the hypocellular component. On the other hand, at the moment of the surgical bleb revision, 2 blebs presented a thick connective tissue suggesting a predominance of the fibrous mechanism.

Regarding dysesthetic blebs baseline characteristics, in our study the 4 cases presented blebs that were not so high but very wide, on average 2.5 × 3 according to IBAGS scale [6]; 3 of 4 eyes had a prominent nasal extension and 2 of 4 eyes had corneal Dellen (Figure 1 (a–d)). This is in accordance with some studies that have looked into which bleb features are more likely to cause ocular discomfort and that have found that although dysesthesia is not significantly associated with bleb height [19]; pain does occur more often in larger and circumferential blebs with intrapalpebral exposition and corneal expansion [3], especially if corneal Dellen and a more nasal location are present [3, 19].

Many treatments have been described to manage this condition. Aggressive surface lubrication is an initial, widely accepted, conservative measure that brings only temporary relief to most cases. Other nonsurgical approaches like topical trichloroacetic acid and injection of autologous blood can be used to reduce bleb height by stimulating scarring, but they can also result in filtration failure [2, 5]. Furthermore, laser applications have been suggested but may require multiple sessions with subsequent inflammation and less predictable IOP control [2]. Corneal Dellen is an uncommon complication of extensive blebs [2, 20] and its satisfactory treatment is extremely important to prevent corneal ulceration and subsequent potential perforation [2]. If lubrication is ineffective, a contact lens (conventional or bandage) can be used [21] but its adequate fitting is often challenging in these cases due to bleb-related conjunctival irregularity [22], so definitive reductive surgery should be taken into account.

In cases of highly symptomatic blebs, surgery is the only effective treatment. Globally, in our systematic review we found a complete surgical success rate (without hypotensive medication) between 43% [11] and 100% [10, 14, 16] depending on the technique used (see Table 2). The worse surgical results were observed in partial or entire bleb excision with advancement of conjunctiva as in Canut et al. [11] (43%), Radhakrishnan et al. [12] (57%), and Catoira et al. [17] (67%) studies. Although this more aggressive approach may be adequate for bleb leaks repair (success rate of 83% [11]), our literature evaluation suggests that performing bleb removal may be counterproductive in dysesthetic blebs in which IOP is well controlled, very likely due to the excessive elimination of conjunctival tissue that was actually working properly. This reasoning supports the idea that in bleb dysesthesia the problem is an excessive size rather than a bad function favoring techniques that preserve bleb conjunctiva.

There are several methods for limiting bleb size without removing conjunctival tissue. Bleb compression sutures have been used as an effective alternative [5, 7, 8], but despite having a success rate of 100% in Faingold and Kasner study [8], 3 out of 4 patients experienced complications (2 reoperations and one bleb leak). “Bleb window”-pexy is a different minimally invasive procedure that is able to reduce the bleb with success rates between 89% [9] and even 100% [10] and a low rate of complications. Another widely used surgical approach is bleb-limiting conjunctivoplasty [18] that has shown excellent outcomes with complete success rates of more than 90% possibly being less effective in blebs with thicker walls and denser appearance [3]. With this hypothesis, Lloyd et al. added conjunctival dissection and further excision of any subjacent subconjunctival tissue to all their procedures (*n* = 13) obtaining very good results (100% success) but still with 3 cases needing additional hypotensive topical treatment [4]. Encouraged by these outcomes, we decided to perform a bleb-limiting conjunctivoplasty as elective surgical treatment for our patients, but deciding intraoperatively to add removal of subconjunctival fibrous tissue only in the cases in which it was clearly identifiable at the time of the procedure (*n* = 2/4, 50%) with the idea to individualize treatment depending on the most predominant causing mechanism. In all of our four cases, discomfort relief and good IOP control were achieved and maintained after one year of follow-up without any additional glaucoma treatment or significant glaucomatous deterioration.

Our study has several limitations. First, due to the rarity of the condition, a small number of cases were included in the study, and this needs to be considered when interpreting the results. And second, our systematic review could not find enough reliable and prospective information to provide more robust data. To partly address these limitations, a thorough and comprehensive evaluation and summary of the available data in the literature was provided.

In conclusion, bleb dysesthesia is an uncommon complication that very likely needs surgery due to failure of medical treatment. Aggressive surgical techniques removing bleb tissue may be less effective in terms of subsequent IOP control. A modified bleb-limiting conjunctivoplasty technique may be a treatment for bleb dysesthesia and allows deciding intraoperatively which mechanisms, avascular or fibrous, predominate, choosing then the most appropriate procedure of bleb reduction, with or without excision of subconjunctival fibrous tissue in each case. After 12-month follow-up, this technique provided dysesthesia relief with good IOP control in the four studied cases.

## Figures and Tables

**Table 1:** Demographic characteristics, dysesthetic bleb characteristics, and surgical outcomes of series of consecutive cases.

	Case 1	Case 2	Case 3	Case 4
*Demographic characteristics*				
Sex/age	M/69	M/69	F/72	F/81
Type of glaucoma	POAG	POAG	POAG	POAG
IOP (mmHg)	21	17	18	16
Topical treatment	PG + *β* + *α*	PG + *β* + *α*	PG + CAI + *α*	PG + *β* + CAI
BCVA	20/50	20/32	20/40	20/40
Primary procedure	PT + MMC	PT + MMC	PT + MMC	PT + MMC
Additional treatment	LSL#3 + 5FU#7	LSL#1 + 5FU#2	LSL#1 + 5FU#1	No
BCVA	20/25	20/25	20/40	20/40
IOP (mmHg)	9	10	6	12
VCDR	0.85	0.95	0.9	0.8
OCT	50	47	52	54
VF-MD (dB)	−16.75	−23.3	−30.44	−28.10

*Dysesthetic bleb characteristics*				
Elapsed time (mo) from primary surgery	13	11	4	9
IBAGS	2(H) × 3(E)	2(H) × 3(E)	3(H) × 3(E)	3(H) × 3(E)
Extension	110°	180°	200°	180°
Signs	Red eye	Red eye	Red eye Dellen	Red eye Dellen
Symptoms	FBS	FBS	FBS	FBS
Lubrication topical treatment	SH 1gtt/h + C 1app/*n*	SH 1gtt/h + C 1app/*n*	SH 1gtt/h + C 1app/*n*	SH 1gtt/h + C 1app/*n*
Elapsed time (mo) to bleb reduction	2	1	1.5	3

*Surgical outcomes*				
Type of surgery	CP + E	CP	CP	CP + E
Signs/symptoms	—/—	—/—	—/—	—/—
IOP 24 hrs (mmHg)	12	10	17	26
IOP 1 mo (mmHg)	10	8	10	14
IOP 6 mos (mmHg)	12	10	10	14
IOP 12 mos (mmHg)	12	10	10	14
VCDR	0.85	0.95	0.9	0.8
OCT	50	52	54	56
VF-MD (dB)	−13.25	−23.85	−30.51	−28.97
BCVA	20/25	20/25	20/40	20/40

M: masculine, F: feminine, POAG: primary open-angle glaucoma, IOP (mmHg): intraocular pressure (millimeters of mercury), PG: prostaglandin analogues, *β*: beta blocker, *α*: alpha adrenergic agonist, CAI: carbonic anhydrase inhibitor, BCVA: best-corrected visual acuity, PT: phaco + trabeculectomy, MMC: mitomycin C, LSL: laser suturolysis, 5FU: 5 fluorouracil, VCDR: vertical cup to disc ratio, OCT: optical coherence tomography, VF-MD(dB): visual field mean deviation (decibels), mos: months, IBAGS: Indiana Bleb Appearance Grading Scale [6], H: height, E: horizontal extent, FBS: foreign body sensation, SH 1gtt/h: sodium hyaluronate 1drop every hour, C 1app/n: carbomer 1 application every night, CP + E: conjunctivoplasty + excision of subconjunctival connective tissue, and CP: conjunctivoplasty.

**Table 2:** Literature review of surgical management of circumferential, dysesthetic bleb after glaucoma filtering surgery.

Study	Type	Sex	Age	*N*	Primary proc	Time to Sx	Type of Sx	Comp ReSx	Presx IOP	IOP 24 h	IOP 1 m	IOP 3 m	IOP 6 m	IOP 12 m	Final IOP	Success (%)^*∗*^	Meds	F-U
S Begum et al. [5]	Retro case series	6F 1M	67 (60–77)	7	T + MMC	18 m	Bleb compression sutures, autologous blood	2 needlings	11 ± 2.7	N/A	11 ± 2.5	11.1 ± 2.5	N/A	12 ± 4.7	12 ± 4.7	N/A	0	29.4 ± 23.7 m
JE Morgan et al. [7]	Retro case series	5F 6M	68 (47–78)	11	T	461 d (41–2023)	Bleb compression sutures, autologous blood	8 autologous blood	6.7 (0–12)	17.3 (9–25)	N/A	N/A	N/A	N/A	11.3 (5–16)	N/A	0	45 w (8–81)
D Faingold et al. [8]	Retro rev	N/A	N/A	4	T + MMC/T + MMC + P	3 ± 2 m	Transconj trab flap suturing	2 resx, 1 leak	4.2 ± 0.6	N/A	N/A	N/A	N/A	N/A	8. 7 ± 3.5	100%	N/A	40.7 ± 17.5 m
IC l-Harazi et al. [9]	Retro rev	4F 5M	71 (56–81)	9	T		Bleb window cryopexy	1 misdirection	13.9 (8–26)	N/A	N/A	N/A	N/A	N/A	13.4 (10–16)	89%	1	15.5 m (5–26)
R Tabet et al. [10]	Retro rev	5F 1M	57.1 ± 12.4	6	4 T + MMC, 2 T + 5FU	1.6 ± 1.8 y	“Bleb window”-pexy	No	9.8 ± 4.4	10.33 ± 4.4	10.0 ± 4.7	9.5 ± 4.4	11 ± 6.2		12.2 ± 2	100%	0	6.6 ± 1.7 m (3.5 to 8.3)
MI Canut et al. [11]	Retro case series	3F 4M	63 (44–78)	7	T + MMC, T + MMC+, NPDS + MMC + SK-gel + P, NPDS + SK-gel	32 (24–108) m	Partial bleb excision + conj advancement	1 filtering sx	14.0 (8–18)	14.8 ± 3.5	14.9 ± 3	N/A	N/A	15.6 ± 6.1	N/A	43% (3/7)	4	43.7 ± 29.9 m
S Radhakrishnan et al. [12]	Retro case series	90F (54%)^*∗∗*^	67 ± 14^*∗∗*^	28	T (69%)^*∗∗*^ + MMC (58%)^*∗∗*^ + P (24%)^*∗∗*^	3.5 ± 3.7 y	Excision of the entire bleb (22) or partial (6) with conj advancement	3 filtering sx, 2 resx, 6 still pain, 3 other (blebitis)	11.9 ± 4.7	N/A	N/A	N/A	N/A	N/A	13.4 ± 5.9	57% (16/28)	3	2.8 ± 2.7 y
CC Schnyder et al. [13]	Retro rev	12F 4M^*∗∗*^	62 ± 15.4^*∗∗*^	2	1 T MMC, 1 T	31 ± 12.7 m	Bleb reduction and free conjunctival autologous graft	1 needling	10 ± 4.2	N/A	N/A	N/A	N/A	N/A	12 ± 2.8	38.3%	0	23 ± 1.4 m
GA Lee et al. [14]	Retro case series	1M	69	1	T + MMC/5FU	N/A	Bleb revision with sliding conj flap and fibrin glue	No	10	N/A	N/A	N/A	N/A	N/A	13	100%	0	31 m
SE LaBorwit et al. [15]	Retro case series	18F 13M^*∗∗*^	57 (14–82)^*∗∗*^	11	8 T/3 T P 5 MMC/2 5FU	2.8 ± 2 y	Bleb reduction (excision with conjunctiva advancement in 10 and conjunctival autograft in 1)	5 resx	11.5 ± 4.3	N/A	N/A	N/A	N/A	N/A	13.2 ± 4.6	N/A	0	18.1 ± 11.7 m
EJ van de Geijn et al. [16]	Retro rev	17F 1M^*∗∗*^	59.6 (21–79)^*∗∗*^	2	T + MMC (75%)^*∗∗*^	14.5 ± 12 m	Bleb excision and conjunctiva and Tenon advancement	No	11 ± 4.2	N/A	N/A	N/A	N/A	N/A	11 ± 0	100%	0	52 ± 11.3 m
Y Catoira et al. [17]	Retro rev	12F 18M^*∗∗*^	45.3 ± 21	3	1 T, 1 T P, 1 T MMC	29.1 m (3–114)	Bleb revision by conj advancement	6.6% ptosis, 13% hypertropia^*∗∗*^	12.7 ± 1.2	N/A	N/A	N/A	N/A	N/A	17 ± 3.5	67%	1	17.3 ± 23.2 m
S Anis et al. [3]	Retro rev	7F 8M	67.6 (51–81)	15	T + MMC/5FU	2.6 ± 3.0 y (3 m-8.6 y)	Bleb-limiting conjunctivoplasty	1 leak resolved spontaneously	9.4 ± 4.7	N/A	9.2 ± 5.0	9.2 ± 5.0	N/A	10.2 ± 6.6	N/A	93.3%	0	>3 m
M Lloyd et al. [4]	Retro rev	3F 8M	60 ± 11.0	13	T + MMC	24 w (11 w-16 m)	Bleb-limiting conjunctivoplasty with removal of subconj scar tissue	No	10.6 ± 3.4	N/A	12.8 ± 5.3	11.8 ± 4.8	11.5 ± 2.7	12.6 ± 3.1	N/A	100%	3	>1 y
R Rahman et al. [18]	Case series	2F	67 ± 5.7	4	2 T	24 ± 17 m	Bleb-limiting conjunctivoplasty	1 resx	12 ± 4.4	N/A	N/A	N/A	N/A	N/A	14 ± 4.2	N/A	N/A	9.5 ± 3.5 m

*N*: number of eyes included, primary proc-primary procedure, sx: surgery, Comp: complications, resx: resurgery, presx: presurgery, IOP (mmHg): intraocular pressure (millimeters of mercury), preop.: preoperatively, Meds: antiglaucoma medications, F-U: follow-up, retro: retrospective study, rev-revision, F: feminine, M: masculine, T: trabeculectomy, MMC: mitomycin C, P: phacoemulsification, 5FU: 5 fluorouracil, NPDS: nonpenetrating deep sclerectomy, d: days, w: weeks, m: months, y: years, N/A: not available. ^*∗*^D Faingold et al. A complete success was defined as achieving a total resolution of the choroidal effusions or a total resolution of symptoms related to dysesthesia without the addition of IOP-lowering drops. ^*∗*^IC l-Harazi et al. Success was defined as subjective relief of symptoms, adequate control of the IOP (no greater than 16 mm Hg), and restoration of filtering bleb function without further antiglaucoma medications or surgical bleb revision. ^*∗*^R Tabet et al. Complete success was defined as complete resolution of symptoms and flattening of the interpalpebral portion of the bleb while maintaining an IOP between 8 and 20 mm Hg without further antiglaucoma medications or surgical bleb revision. ^*∗*^MI Canut et al. Complete success defined as maintenance of individual target IOP without a second revision, surgery, or glaucoma medications. ^*∗*^S Radhakrishnan et al. Successful outcome was defined as elimination of primary indication, no requirement for further intraocular pressure- (IOP-) lowering surgery, no major complication, and no development of new bleb-related complication. ^*∗*^CC Schnyder et al. The complete success rate was defined by an IOP >6 mm Hg and <21 mm Hg, with a visual acuity equal to or better than the preoperative visual acuity without any glaucoma medication. ^*∗*^GA Lee et al. A successful outcome was defined as the resolution of the presenting indication for revision, with maintenance of IOP with the same or reduced number of glaucoma medications in the absence of further glaucoma surgery. ^*∗*^EJ van de Geijn et al. Surgical success was defined as a final intraocular pressure between 6 and 22 mm Hg with or without topical antiglaucoma medication, resolution of symptoms, and no need for repeat glaucoma surgery (except for repeat revision surgery). ^*∗*^Y Catoira et al. Success was defined as resolution of the bleb-associated complication necessitating the revision (discomfort) with maintenance of intraocular pressure greater than or equal to 6 and less than or equal to 21 mm Hg without glaucoma medications. ^*∗*^S Anis et al. Success criteria were defined as subjective resolution of symptoms and maintenance of IOP with no subsequent surgical intervention. ^*∗*^M Lloyd et al. Bleb functionality defined as adequate IOP control without further surgery. ^*∗∗*^The whole studied cohort.

## Data Availability

All data relevant to this study are included within the article or uploaded as online supplementary information. The data are also available from the corresponding author upon reasonable request (agnieszkaannadyrda@wp.pl).

## Supplementary Materials

Supplementary Materials The supplementary file is the video explaining the surgical method discussed in the article.
